# Compulsory Interventions in Severe and Persistent Mental Illness: A Survey on Attitudes Among Psychiatrists in Switzerland

**DOI:** 10.3389/fpsyt.2021.537379

**Published:** 2021-05-25

**Authors:** Julia Stoll, Martina A. Hodel, Florian Riese, Scott A. Irwin, Paul Hoff, Nikola Biller-Andorno, Manuel Trachsel

**Affiliations:** ^1^Institute of Biomedical Ethics and History of Medicine, University of Zurich, Zurich, Switzerland; ^2^Psychiatric Clinic Zugersee, Zug, Switzerland; ^3^Psychiatric University Hospital Zurich, Zurich, Switzerland; ^4^Cedars-Sinai Health System, Los Angeles, CA, United States; ^5^Clinical Ethics Unit, University Hospital Basel and University Psychiatric Clinics Basel, Basel, Switzerland

**Keywords:** compulsory interventions, coercion, paternalism, autonomy, psychiatry, ethics, severe and persistent mental illness, palliative care

## Abstract

**Background:** Some psychiatric patients develop severe and persistent mental illness (SPMI), which, for a variety of reasons, can be therapy-refractory. Sometimes, treatment is not considered helpful by the patients themselves and does not improve their subjective quality of life. Furthermore, many SPMI patients experience compulsory interventions such as seclusion, restraint, or treatment against their will, which can cause harm.

**Methods:** In a cross-sectional survey of 1,311 German-speaking psychiatrists in Switzerland, participants were asked about the care of SPMI patients in general, and about their attitudes with regard to compulsory interventions in particular, using three case vignettes of patients with severe and persistent anorexia nervosa, schizophrenia and depression.

**Results:** Out of 1,311 contacted psychiatrists, 457 (34.9%) returned the completed survey. In general, 91.0% found it important or very important to respect SPMI patients' autonomy in decision making. However, based on three different clinical case vignettes, 36.8% of psychiatrists would act against the wishes of the patient with severe and persistent schizophrenia, 34.1% against the wishes of the patient with severe and persistent depression, and 21.1% against the wishes of the patient with severe and persistent anorexia nervosa, although all patients were stated to have preserved decision-making capacity. With regard to the case vignettes, 41.1% considered compulsory interventions leading to a temporary reduction of quality of life acceptable in the patient with severe and persistent schizophrenia, 39.4% in the patient with severe and persistent depression, and 25.6% in the patient with severe and persistent anorexia nervosa, although it was stated in all three case vignettes that two independent experts ascribed the patients decision-making capacity regarding their illness and further treatment.

**Conclusions:** Many psychiatrists in our sample found themselves in an ethical dilemma between autonomy and the provision of medical care. While most respondents respect the autonomy of SPMI patients, many saw the need to perform compulsory interventions even though it was clearly and prominently stated that two independent psychiatrists had ascribed the patients in the case vignettes decision-making capacity. Further examination of these conflicting views is warranted, perhaps along with the development of guidelines for such situations.

## Introduction

Some patients with psychiatric disorders develop severe and persistent mental illness (SPMI). For the present study, we defined SPMI as a chronic or long lasting and severe mental illness, persisting after several competent treatment attempts and causing significantly impaired functioning in daily life [see (1); for a review of definitions see (2)]. Further, some patients with SPMI might perceive psychiatric treatment as intrusive, and additional treatments might not be helpful or improve an already poor quality of life. In addition, SPMI patients might be more prone to experiencing compulsory interventions [see (3)].

The Swiss Academy of Medical Sciences defines “[c]oercion (the use of force) [as] carrying out a measure in spite of the fact that the person concerned either indicates or has previously indicated – through an expression of wishes or opposition – that he or she does not consent to it” (p. 7). Szmukler and Appelbaum (4) expand this definition by broadening the scope of possible *treatment pressures* experienced by patients, being “(1) persuasion; (2) interpersonal leverage; (3) inducements; (4) threats; [and] (5) compulsory treatment (in the community or as an inpatient)” (p. 234).

In the last 20 years, several survey studies have been conducted focusing on attitudes toward compulsory interventions held by staff working with mentally ill patients, for example in the context of schizophrenia, anorexia nervosa, or inpatient psychiatric emergency situations [see e.g., (5–14)].

As mentioned above, patients with SPMI are particularly prone to intense and intrusive treatment including compulsory interventions that might not necessarily lead to a higher quality of life. Therefore, scholarly discussion of treatment options of patients with SPMI began to focus on new approaches including palliative care [see (15–17)]. Based on the WHO definition of palliative care, a working definition of palliative psychiatry has been proposed by Trachsel et al. (18): “Palliative psychiatry (PP) is an approach that improves the quality of life of patients and their families in facing the problems associated with life-threatening severe persistent mental illness (SPMI) through the prevention and relief of suffering by means of a timely assessment and treatment of associated physical, mental, social, and spiritual needs. PP focuses on harm reduction and on avoidance of burdensome psychiatric interventions with questionable impact” (p. 3). The authors have proposed that the beneficiaries of palliative psychiatry are patients with SPMI, who are at risk of therapeutic neglect and/or overly aggressive care within current paradigms. In a survey on which also the present study is based, attitudes of psychiatrists toward such a palliative approach have been assessed [see (17)].

Notwithstanding the above-mentioned discussion of palliative care in patients with SPMI, it was the aim of the present study to explore potential conflicts between the ethical goal of respecting patients' autonomy on the one hand and beneficence as well as paternalism on the other hand. Therefore, psychiatrists' attitudes on compulsory interventions in patients with SPMI are the focus of the present article. To our knowledge, to date no other study has particularly focused on attitudes of psychiatrists toward compulsory interventions in patients with SPMI being explicitly stated to having decision-making capacity regarding their further treatment.

## Materials and Methods

A cross-sectional survey of 1,311 German-speaking psychiatrists in Switzerland, all members of the Swiss Society for Psychiatry and Psychotherapy (SSPP), who represent approximately 30% of all psychiatrists in Switzerland, was conducted. The survey covered questions regarding attitudes toward palliative care approaches, toward medical assistance in dying and compulsory treatment of patients with SPMI. After the SSPP informed members about the intent of the study, they received a survey by mail with a prepaid return envelope. The data collection period lasted from February to March 2016. Participants received a reminder postcard 4 weeks after the survey had been mailed.

### Survey and Case Vignettes

The survey in German language consisted of 42 items and an open comment field [see Table 1, see also (17)]. The first five questions about treatment of SPMI patients in general were answered on a 7-point Likert scale ranging from unimportant (0) to very important (6) with a neutral midpoint (3). The following 13 questions regarding palliative care and assisted suicide in patients with SPMI and the next seven questions relating to three case vignettes (see Table 2) were answered on a 7-point Likert scale ranging from completely disagree (−3) to completely agree (+3) with a neutral midpoint (0).

**Table 1:** Survey items [based on (17)].

**1: Questions on the treatment of patients with severe and persistent mental illness (SPMI)**	
In the treatment of patients with SPMI, how important is	(a) curing the illness?
	(b) reduction of suffering?
	(c) the patient's ability to function in daily life?
	**(d) the patient remaining autonomous in their decision making?**
	(e) impeding suicide?
**According to the World Health Organization, palliative care “is an approach that improves the quality of life of patients and their families facing the problem associated with life-threatening illness, through the prevention and relief of suffering by means of early identification and impeccable assessment and treatment of pain and other problems, physical, psychosocial and spiritual.”**	
How strongly do you agree or disagree with the following?	(f) For me, the term “palliative” relates directly to end of life.
	(g) For some SPMI patients, palliative care is indicated.
	(h) In psychiatry, applying a palliative care model is important in providing optimal support for certain patients without a life-limiting medical illness.
	(i) In severe, chronic and therapy-refractory anorexia nervosa, a palliative approach would be suitable.
	(j) In severe, chronic and therapy-refractory schizophrenia, a palliative approach would be suitable.
	(k) In severe, chronic and therapy-refractory depression, a palliative approach would be suitable.
	(l) In severe, chronic and therapy-refractory bipolar disorder, a palliative approach would be suitable.
	(m) In severe, chronic and therapy-refractory substance disorder, a palliative approach would be suitable.
How strongly do you agree or disagree with the following?	(n) SPMI can be a terminal illness.
	(o) Sedation for the reduction of unbearable refractory psychological symptoms is justifiable in certain cases of SPMI.
	(p) I would generally be willing to perform sedation as mentioned in item o.
	(q) I generally advocate for the access to assisted suicide for patients with SPMI.
	(r) I would generally support a patient in his or her wish to seek assisted suicide by writing a medical report or referring him or her to a respective organization.
**2: Questions about the three case vignettes**	
Please evaluate the three case vignettes.	(a) I would not be surprised if this patient died within the next 6 months.
	(b) For this patient, further curative interventions would most likely be futile.
	(c) In this case, I would be comfortable with a reduction of life expectancy to increase or maintain quality of life if consistent with the patient's goals.
	**(d) In this case, I would accept a temporary decrease in quality of life because of coercive measures**.
	**(e) In this case, I would not proceed against the patient's wishes**.
	(f) In this case, sedation to reduce an unbearable refractory symptom is reasonable.
	(g) If there is an explicit and enduring wish for assisted suicide, I would support this patient in his or her plan and refer him or her to a respective organization.

*The Table has been designed in the style of Hodel et al. (19) and Trachsel et al. (17). The items relevant for this article are tagged in **bold***.

**Table 2:** Case vignettes.

**Case 1:** 37-year-old female with anorexia nervosa, onset at age 11	
	Symptoms: general muscle weakness; loss of bone density; amenorrhea; current weight 24 kg/52 lb; body mass index 9.5 kg/m^2^; no recent weight gain or stabilization; no acute danger of dying as her body is adapted to being underweight. The patient underwent 10 previous inpatient treatments (in both somatic and psychiatric hospitals), three of which were in specialized psychiatric institutions. Throughout the course of disease, different intensive psychotherapies have been tried without success. During hospitalizations, the patient underwent several artificial refeedings, sometimes under sedation. The patient now refuses artificial refeeding and treatment. She states that, for years, her life has been focused exclusively on trying to overcome her anorexia, leaving her without friends or hobbies. She suffers from physical symptoms, including general muscle weakness and loss in bone density, saying that she would rather die than undergo further treatment and wishes to be left in peace. She does not want to be forced into eating anymore. Two experts have declared that the patient has decision-making capacity to refuse further treatment, with consequent risk of dying.
**Case 2:** 33-year-old male with schizophrenia, onset at age 17, no significant comorbidities	
	Positive symptoms: auditory and visual hallucinations, persecutory delusions. Negative symptoms: apathy, social withdrawal, poverty of speech (all rated severe). Despite long-lasting, high-dose pharmacological treatment (several atypical neuroleptics, haloperidol, clozapine, and combinations of these), as well as electroconvulsive therapy, the patient has never been free from positive or negative symptoms. Multiple psychotherapies of various kinds have also failed to stabilize the patient or to improve his quality of life. He does not wish to continue assertive community treatment because he feels it is too intrusive. Although the positive symptoms were more dominant in the first years following initial diagnosis, he went on to develop severe negative symptoms, as well as aggression and self-injurious behavior such as burning himself with cigarettes. The negative symptoms and his strong functional deficits are exacerbated by chronic unemployment and inability to live independently; the patient has no family system. His persisting illness has left him completely isolated, with no social contacts and no hobbies or interests. Two experts have declared that he possesses decision-making capacity in respect of his illness and its treatment.
**Case 3:** 40-year-old male with major depressive disorder, no significant comorbidities	
	Symptoms: energy loss, insomnia, fatigue, persistent suicidal ideation over 20 years, current acute and concrete suicidal intent. The patient underwent different intensive, evidence-based, long-term psychotherapies, including specialized treatment approaches such as cognitive behavioral analysis system of psychotherapy (CBASP) and interpersonal psychotherapy (IPT). His depression was not improved either by psychotherapy alone or in combination with adequate treatment trials of antidepressants (selective serotonin reuptake inhibitors, tricyclic antidepressants, venlafaxine, augmentation with lithium and antipsychotic medications [quetiapine and aripiprazole]). The patient experienced significant adverse effects with several of the medications. Exhausted and as a last resort, he has decided to undergo electroconvulsive therapy. However, maintenance electroconvulsive therapy proved equally ineffective in preventing the reappearance of suicidal ideation; indeed, the symptoms worsened. The patient experiences severe hopelessness and states that his quality of life is very poor, that he doesn't want to deal with his illness anymore, and that he plans to commit suicide in the near future. Two experts have declared that he possesses decision-making capacity regarding his illness and its treatment.

*Case vignettes modified from Brenner et al. (20), Baweja and Singareddy (21), and Trachsel et al. (22) and depicted in style of Hodel et al. (19) and Trachsel et al. (17)*.

In order to harmonize the three case vignettes regarding the criteria of futility, they have been chosen and adjusted with regard to this criterion from previously published materials [see (20–22)]. Furthermore, they have been adjusted in order to make them more comparable regarding preserved decision-making capacity, age, gender, and applied therapy options.

The usability of the questionnaire was tested with a pilot sample of 10 psychiatry residents.

### Statistical Analysis

For age and work experience, arithmetic means were calculated, and for the other variables regarding attitudes toward patient's autonomy, acting against the patient's wishes, and compulsory interventions, descriptive statistics (percentages) were calculated. The two 7-point Likert scales were aggregated to three categories indicating disagreement/unimportance (−3, −2, −1/0, 1, 2), neutrality (0/3), or agreement/importance (1, 2, 3/4, 5, 6). Statistical analysis was conducted using IBM Statistics Version 25.

To take a closer look at the distribution of the variables “age” and “work experience,” median values were calculated and the Shapiro-Wilk-test for normal distribution was performed. An additional exploratory analysis of the correlation between age and the questionnaire item “In this case, I would accept a temporary decrease in quality of life because of coercive measures.” was conducted using the Spearman correlation since the data of the questionnaire item was not normally distributed in all three case vignettes (Shapiro-Wilk-test *p* < 0.05).

A second exploratory analysis was conducted regarding the influence of the psychiatric diagnosis depicted in the case vignette on the responses given regarding the items “In this case, I would accept a temporary decrease in quality of life because of coercive measures.” and “In this case, I would not proceed against the patient's wishes.” The analysis was conducted using the Kruskal-Wallis test, because the questionnaire items were not normally distributed in all three case vignettes (Shapiro-Wilk-test *p* < 0.05) [see (23)]. In a second step, a follow-up analysis using multiple Mann-Whitney U-tests with Bonferroni correction for correcting the *p*-value for multiple testing was used to determine which items of which diagnosis were significantly different from each other [see (24)]. For further specifying the strength of these differences, effect sizes were calculated for each pairwise comparison [see (23)].

For verification purposes, the study data set is available from the corresponding author upon request.

### Ethical Statement

For the present study, no personal data concerning human disease and the function or structure of the human body was collected. Therefore, the study was outside the scope of the Swiss Human Research Act. The study protocol had been evaluated according to the Checklist for the ethical evaluation of empirical studies that do not need mandatory authorization (CEBES; no. CEBES-2016-04) of the Institute of Biomedical Ethics at the University of Zurich, Switzerland. The participant data were collected anonymously. In order to guarantee anonymity, no informed consent document was used and no incentives were provided. Completing and returning the questionnaire was considered implicit consent for study participation.

## Results

### Sample Characteristics

Of the 1,311 contacted psychiatrists, 457 (34.9%) returned a completed survey. The mean age of the participants was 57.7 years (4.4% missing) with a median age of 58.0 years, and according to the Shapiro-Wilk-test (p > 0.05), the variable “age” was normally distributed. 58.9% were male (4.2% missing). Respondents had a mean work experience of 27.7 (5% missing data) with a median work experience of 27.0 years after graduation from medical school showing a slightly right-skewed distribution (Shapiro-Wilk-test *p* < 0.05). Three participants had a work experience after graduation from medical school higher than 50 years.

### Psychiatrists' Attitudes on Respecting the Patients' Autonomy

Participants were asked how important they perceived the autonomous decision making of patients with SPMI. 91.0% found it important or very important, 1.5% found it less important, and 6.8% remained neutral (0.7% missing) (see Table 3).

**Table 3:** Results regarding psychiatrists' attitudes on respecting the patients' autonomy.

	**Less important**	**Neutral**	**Important or very important**	**Missing**
Respecting the patients' autonomy	1.5%	6.8%	91.0%	0.7%

### Psychiatrists' Attitudes on Acting Against the Patients' Wishes

**Figure 1:**
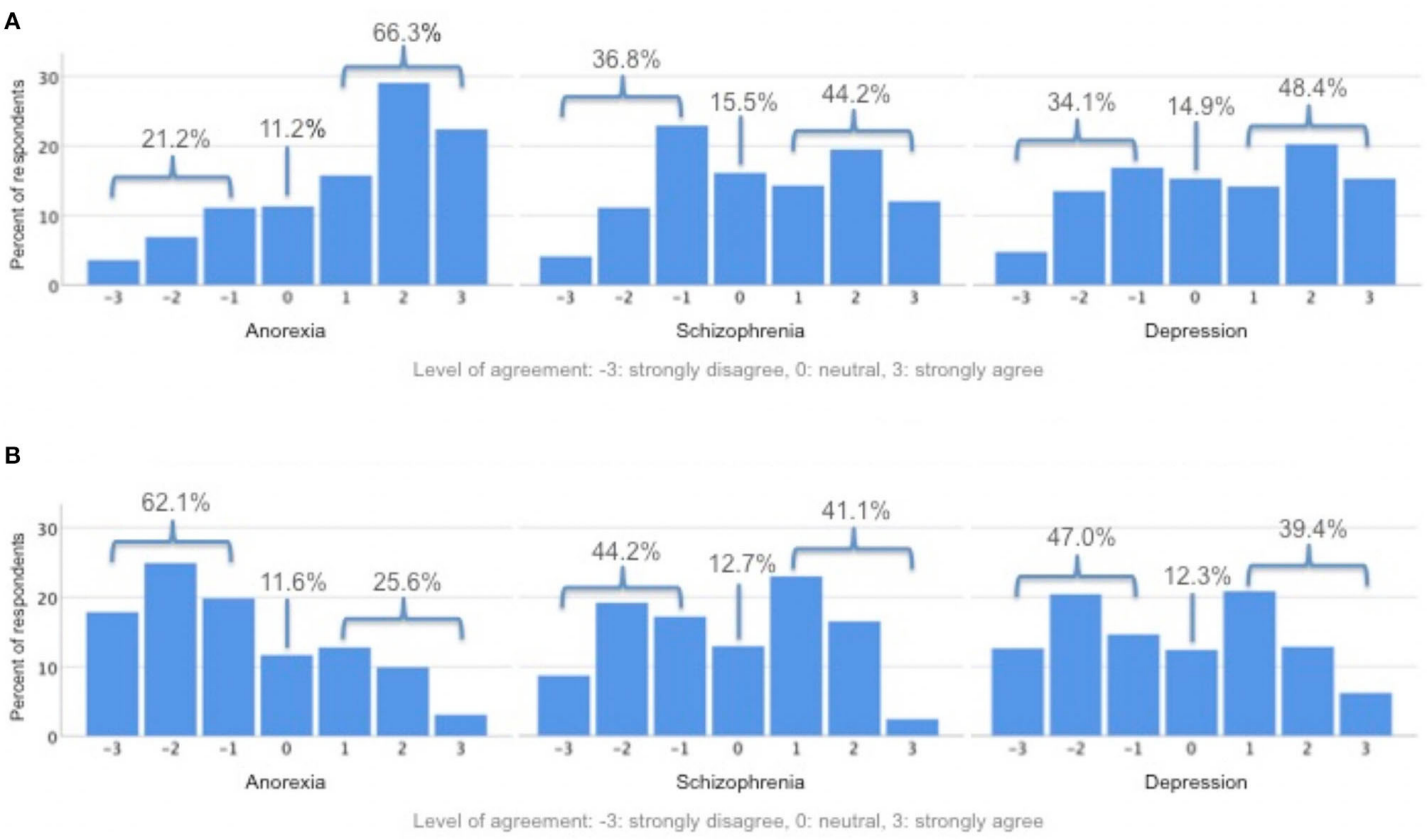
**(A)** In this case, I would not proceed against patient's wishes. **(B)** In this case, I would accept a temporary decrease in quality of life because of coercive measures.

With regard to the three case vignettes, participants were asked if they would act against the wishes of the respective patient. 21.1% indicated that they would act against the wishes of the patient with severe and persistent anorexia nervosa, 66.3% would not, and 11.2% remained neutral (1.3% missing). 36.8% indicated that they would act against the wishes of the patient with severe and persistent schizophrenia, 44.2% would not, and 15.5% remained neutral (3.5% missing). 34.1% indicated that they would act against the wishes of the patient with severe and persistent major depressive disorder, 48.4% would not, and 14.9% remained neutral (2.6% missing) (see Figure 1).

### Psychiatrists' Attitudes on Compulsory Interventions in Patients With SPMI

With regard to the three case vignettes, participants were asked if they would use compulsory interventions, accepting a temporary reduction of quality of life. 25.6% indicated that they would use compulsory interventions in the case of the patient with severe and persistent anorexia nervosa, 62.1% would not, and 11.6% remained neutral (0.7% missing). 41.1% indicated that they would use compulsory interventions in the case of the patient with severe and persistent schizophrenia, 44.2% would not, and 12.7% remained neutral (2.0% missing). 39.4% indicated that they would use compulsory interventions in the case of the patient with severe and persistent major depressive disorder, 47.0% would not, and 12.3% remained neutral (1.3% missing) (see Figure 1).

### Exploratory Analysis: Age and Psychiatrists' Attitudes on Compulsory Interventions

A negative Spearman correlation between age and the questionnaire item “In this case, I would accept a temporary decrease in quality of life because of coercive measures.” was found for all three case vignettes (anorexia nervosa: r_s_ = −0.158, *p* = 0.001, *n* = 434; schizophrenia: r_s_ = −0.149, *p* = 0.002, *n* = 428; depression: r_s_ = −0.172, *p* < 0.001, *n* = 431).

### Exploratory Analysis: Psychiatric Diagnosis and Psychiatrists' Attitudes on Compulsory Interventions and Proceeding Against the Patient's Wishes

Using the Kruskal-Wallis test the answers to the item “In this case, I would accept a temporary decrease in quality of life because of coercive measures.” [*H*_(2)_ = 34.29, *p* < 0.001] and the item “In this case, I would not proceed against the patient's wishes.” [*H*_(2)_ = 47.23, *p* < 0.001] were overall significantly different in regard to the psychiatric disorder depicted in the case vignettes.

Pairwise comparison of the answers to the items regarding the different case vignettes using the Mann-Whitney U-test with corrected *p*-values using Bonferroni correction (p') showed the following: Regarding the item “In this case, I would accept a temporary decrease in quality of life because of coercive measures.” the psychiatrists disagreed significantly more in the case vignette depicting the patient with severe and persistent anorexia nervosa (Mdn = −1.00) than to the case vignettes depicting the patient with severe and persistent schizophrenia (Mdn = 0.00; *U* = 80065.50, *z* = −5.61, *p* < 0.001, *r* = −0.19) and severe and persistent major depressive disorder (Mdn = 0.00; U = 85498.50, *z* = −4.36, *p* < 0.001, *r* = −0.14). The answers to this item were not significantly different comparing the case vignettes depicting a patient with severe and persistent schizophrenia and severe and persistent major depressive disorder (*U* = 97253.50, *z* = −0.983, *p* = 0.978, *r* = −0.03). The psychiatrists agreed significantly more in the case vignette depicting the patient with severe and persistent anorexia nervosa (Mdn = 2.00) than to the case vignettes depicting the patient with severe and persistent schizophrenia (Mdn = 0.00; *U* = 74911.00, *z* = −6.48, *p* < 0.001, *r* = −0.22) and severe and persistent major depressive disorder (Mdn = 1.00; *U* = 80187.50, *z* = −5.29, *p* < 0.001, *r* = −0.18) regarding the item “In this case, I would not proceed against the patient's wishes.” Comparing the answers to this item regarding the case vignettes depicting a patient with severe and persistent schizophrenia and severe and persistent major depressive disorder no statistically significant difference was found (*U* = 94578.00, *z* = −0.943, *p* = 1.035, *r* = −0.03).

## Discussion

Most of the psychiatrists who responded to this survey found it important or very important to respect the autonomy of the patient with SPMI. Nonetheless, 21.1–36.8% would act against the wishes of these patients. 25.6–41.1% would even use compulsory interventions accepting a temporary reduction of quality of life.

These findings are consistent with the perceived ethical dilemma between respecting patients' autonomy and providing beneficial medical care (beneficence) [see (25–27)]. Coercive actions might be justified in the light of the “*patient's best interest*” or benefit (25, 26). Relating the concepts of autonomy and decision-making capacity to the concept of deciding “in the best interest of the patient,” leads to the debate on weak vs. strong paternalism. According to *weak paternalism* “[…] a man can rightly be prevented from harming himself […] if his intended action is substantially non-voluntary or can be presumed to be so in the absence of evidence to the contrary” [25, p. 124]. *Weak paternalism* has also been defined as deciding for a person *without* decision-making capacity in his or her best interest (28, 29). In contrast, *strong paternalism* can be defined as deciding for a person who possesses decision-making capacity (as in the case-vignettes used in this survey). In the words of Feinberg (30), strong paternalism is embraced when a person is protected “[…] against his will, from the harmful consequences even of his fully voluntary choices and undertakings” (p. 124).

Generally speaking, weak paternalism is often morally accepted (28). However, if the patient is able to make autonomous decisions regarding his or her further treatment, i.e., has decision-making capacity, compulsory interventions are regarded as strong paternalism and should not be used and are even prohibited in most jurisdictions [see (25–27, 31)]. In the present survey, it was explicitly stated in the case vignettes that two independent psychiatrists had ascribed decision-making capacity to the patients. Yet, some respondents indicated that they would proceed against the patient's wishes or accept a temporary decrease in quality of life due to coercive measures. This is consistent with findings in Aasland et al. (14) study. They conducted a nationwide survey in Norway among staff working with mentally ill patients using six vignettes from clinical situations where compulsory interventions was among the proposed actions. The authors discovered that some participants chose illegal actions. None of the scenarios is directly comparable to our three vignettes, but in one scenario describing a suicidal patient (claimed as not suffering from a serious mental disorder), one proposed action was to “admit the patient to coerced observation” (p. 108) which was considered illegal in Norway. Still 38.8% of the psychiatrists chose this option – a comparable figure to our findings (ranging from 21.2 to 36.8%, respectively 25.6 to 41.1%). Just as Aasland et al. (14), we do not know whether the proposed treatment in our survey is a conscious decision to disagree with the law, reflects ignorance of the regulations, or is a lack in knowledge of the law itself. Aasland et al. (14) speculate that the regulation itself might be too complicated and unclear. However, it should be noted that in the present survey, no direct question about the explicit action of the psychiatrist was asked. In other words, they were not asked specifically whether they would actually carry out coercive measures in these cases.

Another possible explanation might be that the psychiatrists doubted the decision-making capacity of the patients, or more specifically, the ascription of decision-making capacity by their colleagues. However, on the one hand, research has shown that decision-making capacity is generally rated with a good level of consistency among different clinicians (32). On the other hand, it has been argued that “[c]linicians might have a tendency to equate treatment refusal with incapacity and treatment acceptance with capacity” [28, p. 295]. The latter is a possible explanation of our findings since all patients described in the case vignettes refused further treatment. In addition, higher illness severity and specifically psychosis were more often found in patients ascribed with decision-making incapacity (33). This might explain the higher rate of accepting compulsory interventions in the patient with severe and persistent schizophrenia (41.1%) compared to the patient with severe and persistent anorexia nervosa (25.6%).

In a survey study, Tan et al. (11) asked psychiatrists about their attitudes toward, among other things, compulsory treatment and the ability to make treatment decisions in anorexia nervosa patients. They found that many psychiatrists believe that anorexia nervosa patients are limited in their ability to make treatment decisions and that coercive measures must often be used. Based on these findings, the slight deviation of the results for severe and persistent anorexia nervosa from the results for severe and persistent schizophrenia or major depressive disorder with respect to the use of coercive measures or acting against the wishes of the patient appears inconsistent (see Figure 1). A possible explanation for our seemingly contrary data to the ones collected by Tan et al. (11) could be that our case vignette represents the rare case in which the anorexia nervosa patient is attributed with decision-making capacity by independent experts. Tury et al. (34) emphasize in their article that refusal of treatment and lack of disease awareness are often typical aspects of anorexia nervosa patients and describe a “coercion paradox,” where the practitioner or parents are forced to perform coercive measures on the patient, for the patient to get a feeling that they are being cared for and to experience the environment around them as controlled. The case vignette of the patient with severe and persistent anorexia nervosa used here is therefore an exception in that it represents a patient with attested decision-making capacity with regard to her treatment. It could be that in this particular case, this is explicitly highlighted and that most psychiatrists surveyed are therefore correctly less inclined to take coercive measures and act against the patient's wishes.

Central to the conflict between autonomy and providing medical care (beneficence) might be an inner challenge to the “professional identity” of the psychiatrist (27), and more specifically, a feeling of helplessness regarding a decision that might result in unchangeable outcome like the death of the patient - particularly when considering the fact that psychiatrists are trained to prevent suicide (3, 19). This might explain the difference in the psychiatrists' attitudes on compulsory interventions between the patient with severe and persistent anorexia nervosa (25.6%) and the patient with severe and persistent major depressive disorder (39.4%) because in the latter, the patient explicitly states “that he plans to commit suicide in the near future” (see Table 2).

In order to further investigate the differences regarding the possible different responses to the use of coercive measures or the acceptance of the patient's wishes depending on the described psychiatric disorder in the case vignettes, a further exploratory analysis was conducted. It was found that the psychiatrists were less willing to accept a reduction of the quality of life by using coercive measures or to act against the wishes of the patients with severe and persistent anorexia nervosa than they were for patients with severe and persistent schizophrenia and severe and persistent major depressive disorder.

These differences regarding attitudes toward compulsory interventions in different disorders are in line with an observational study in the Psychiatric University Hospital Zurich, Switzerland, where patients with psychotic disorders and risk of harm to self (or others) were more likely to experience compulsory interventions (35). In a German study on alternatives to compulsory interventions, lesser use of alternative actions to compulsory interventions could be mostly explained by the symptoms of the patients like psychotic symptoms, and interestingly the fear of an illegal action in the sense of failure to provide assistance (36). Thus, the feeling of helplessness, the wish for care for the patient, or the fear that refraining from compulsory interventions might lead to an unchangeable outcome, namely, the death of the patient, might constitute central factors explaining the expressed attitudes of the participants in the present study.

### Strengths and Limitations

One strength of this study is that, as far as we know, it is the first study to date which explicitly deals with psychiatrists' attitudes toward coercive measures in patients with SPMI which had been independently assessed by two experts to be capable of decision making. By showing that there are psychiatrists who oppose the decision of a patient stated to having decision-making capacity or at least do not seem to agree that the patient has decision-making capacity although stated by two experts or use coercion anyway, this study sheds light on the relevance and reality of the dilemma between autonomy and beneficence discussed in the literature and above. Further, the study highlights the controversial issue of coercive interventions vs. patient autonomy in the most severely ill; it is a timely paper because it addresses questions related to assisted dying in mental health, and it has a comparatively large sample as many countries do not even have that many psychiatrists. Additionally, Switzerland could overall be considered to have a liberal society, therefore, it is informative in so far that the study documents the current attitudes in such a liberal society.

The results of the present survey might be limited due to a sampling bias because those who responded represent only about 10% of psychiatrists in Switzerland, the sample consisted of only German-speaking psychiatrists [see (17, 19)], and only a third of the contacted psychiatrists actually responded to the survey. With regard to the sample, it should also be noted that it may not consist of a representative group of psychiatrists given their mean age of 57.7 years, their mean work experience of 27.7 years after graduation from medical school, their SSPP membership, and language (German). This could limit the generalizability of the findings. Because of this possible limitation, an exploratory analysis of the data was additionally performed using Spearman correlation. The negative correlation between age and the questionnaire item “In this case, I would accept a temporary decrease in quality of life because of coercive measures.” might indicate that younger psychiatrists are more likely to accept a temporary decrease in quality of life because of coercive measures in relation to the case vignettes. In a future study, it would be interesting to focus on younger psychiatrists.

Additionally, a response bias might be present due to the respondents' particular interest in the topics of compulsory interventions, palliative care or assisted suicide, thus representing a special collective [see (17, 19)]. Using Likert scales for answer options might additionally influence the results since they are unable to capture the nature of complex and multidimensional topics [see (17, 19)].

Another important limitation could be that the findings might not be generalizable to other professionals working with patients with SPMI [see (17)]. Previous studies have found differences between the different professions working with mentally ill patients regarding their attitudes toward the use of compulsory interventions [see (6, 7, 9, 14)]. Hereby, psychiatrists were found to be more favorable regarding the use of compulsory interventions than other medical professionals [see (7, 14)]. One reason for different decision making might be the fact that psychiatrists are commonly decision makers (14) and carry significant responsibility for treatment decisions. This is supported by a finding in a study by Wynn et al. (10). In their study, psychologists who have previously made decisions regarding compulsory interventions were more favorable with regard to the use of compulsory interventions than psychologists not previously involved in the decision process.

Another aspect of the present study is that the case vignettes are not representative with regard to the respective disorder patterns but need to be seen as particularly exceptional cases. This is especially true with regard to the decision-making capacity regarding the patients' further treatment attested by two independent experts. Psychiatrists can vary widely in their assessment of the need for coercive measures [see (37)] which highlights the possibility that some of the surveyed psychiatrists may not have trusted their colleagues' conclusion on the decision-making capacity of those patients with regard to their further treatment. In retrospect, it would have been interesting to shed further light on precisely this point, i.e., to ask participants whether they would have differently evaluated the decision-making capacity of the patients described in the case vignettes. This would be an interesting question for further research.

In the present survey, no definition of “compulsory intervention” was given. Thus, we do not know the concept the participants had in mind answering the survey questions. In addition, informal compulsory interventions were not explicitly included in the survey.

An important further limitation results from how the participants might have interpreted the questions asked in the survey. The chosen wording “not proceed against the patient's wishes” and “would accept a temporary decrease in quality of life because of coercive measures” may have left too much room for interpretation. The participants were not asked for a specific action, for example, “How likely would you be to use coercive measures in this case?” Therefore, the results must be carefully interpreted, and no direct conclusions can be drawn about the specific and explicit intention of the psychiatrists surveyed.

Finally, it has to be mentioned that the data collection was in 2016 and therefore, the presented data might not exactly reflect the opinions of currently working psychiatrists.

### Future Research and Implications for Clinical Practice

With regard to the above discussed limitations of the present study, it might be worthwhile to conduct another survey including participants from different professions working with patients with SPMI. Additionally, a more distinct definition of compulsory interventions might be provided such as the one suggested by Szmukler and Appelbaum (4). Furthermore, informal compulsory interventions could be included in order to investigate the potential use of interventions commonly underestimated by practitioners [see (38)]. Regarding the ambivalent attitude findings in this survey, further research in the field of decision-making capacity assessment and consensus between practitioners, e.g., how much the evaluation of another clinician is trusted, might be interesting. In a future study, the experts' assessment of the patient's decision-making capacity could be randomized in the case vignettes, i.e., that the psychiatrists would randomly be presented with a case vignette with attestation of decision-making capacity by two independent experts or the same case vignette without attestation of decision-making capacity. This would allow a more nuanced study of the influence of this statement on the psychiatrists' responses with regard to compulsory interventions (paternalism) or respecting the patients' autonomy.

Future studies could further examine the differences found here in the exploratory analysis regarding psychiatric disorders and the use of coercive measures or acting against the patient's wishes, specifically when these patients are attested of having decision-making capacity or not.

For further studies, it might be also worthwhile to focus specifically on younger psychiatrists and their attitudes toward compulsory interventions. Our findings highlight that the topic of compulsory interventions and their legal and ethical limits need to be included in the education of future psychiatrists. A particular focus should be given to the ethical dilemmas between the principles of respect for autonomy and the provision of medical care (beneficence), as well as the specific practical impact on psychiatrists' every day decisions [see (27)]. It seems vital that these conflicts are further discussed in the literature, and guidelines regarding clinical practice need to be established. To help professionals with their clinical decisions in such difficult, often ambivalent, situations, clinical ethics support services might also be useful [for clinical ethics support in mental health care, see e.g., (39–42)].

## Conclusion

The present study highlights the importance of the awareness of ethical aspects in psychiatrists' daily practice – especially with regard to the multidimensional topic of compulsory interventions in patients with SPMI. Cassell (43) wrote in his article about the nature of suffering: “Even in the best settings and with the best physicians, it is not uncommon for suffering to occur not only during the course of a disease but also as a result of its treatment” (p. 639). Psychiatrists should be aware of this and thoroughly weigh the suffering caused by treatments, including compulsory interventions, the relief of suffering which sometimes might include a palliative approach, and respecting the autonomy of patients.

## Data Availability Statement

The datasets generated for this study are available on request to the corresponding author.

## Ethics Statement

The studies involving human participants were reviewed and approved by Checklist for the ethical evaluation of empirical studies that do not need mandatory authorization (CEBES), Institute of Biomedical Ethics, University of Zurich, Switzerland. Written informed consent for participation was not required for this study in accordance with the national legislation and the institutional requirements.

## Author Contributions

JS and MT analyzed the data and drafted the manuscript. MT and MH constructed the questionnaire and conducted the survey. MH, FR, SI, PH, NB-A, and MT were involved in the study conception and design. All authors were involved in the interpretation of the data, the critical revision of the drafted manuscript, and approved the final version submitted for publication.

## Conflict of Interest

The authors declare that the research was conducted in the absence of any commercial or financial relationships that could be construed as a potential conflict of interest.
